# Identification of predictors for the comprehensive clinical risk and severity of coronary lesions of acute coronary syndrome

**DOI:** 10.3389/fcvm.2023.1046895

**Published:** 2023-04-06

**Authors:** Lihui Li, Guangfeng Sun, Jiangbo Yu, Gaojun Shan, Lide Su, Guo Dong

**Affiliations:** ^1^ Department of Cardiovascular, First Affiliated Hospital of Harbin Medical University, Harbin, China; ^2^ Department of Emergency, Xiamen Cardiovascular Hospital, Xiamen University, Xiamen, China

**Keywords:** predictor, acute coronary syndrome, coronary artery disease, GRACE score, SYNTAX score

## Abstract

**Background:**

Acute coronary syndrome (ACS) is the most common cause of death in patients with coronary artery disease. The aim of the study was to identify the predictors of both comprehensive clinical risk and severity of coronary lesions by comprehensive use of GRACE and SYNTAX scores in patients with ACS.

**Methods:**

Clinical data of 225 ACS patients who underwent coronary angiography between 2015 and 2016 were collected. Multiple logistic regression analysis (stepwise) was used to identify the predictors. The predictive ability of predictors and the model were determined using receiver operating characteristics analyses.

**Results:**

Multivariable logistic regression analyses showed that high aspartate aminotransferase (AST) predicted the comprehensive clinical risk with odds ratios (ORs) and 95% confidence intervals (CIs) of 1.011 (1.002–1.021). High total cholesterol (TC) and red blood cell distribution width (RDW) predicted the severity of coronary lesions with ORs and 95% CIs of 1.517 (1.148–2.004) and 1.556 (1.195–2.028), respectively. Low prealbumin predicted both severity of coronary lesions and comprehensive clinical risk of ACS patients with ORs and 95% CIs of 0.743 (0.672–0.821) and 0.836 (0.769–0.909), respectively. The model with a combination of prealbumin and AST had the highest predictive efficacy for comprehensive clinical risk, and the combination of prealbumin, TC, and RDW had the highest predictive efficacy for the severity of coronary lesions. The sensitivity and specificity, and the optimal cut-off values of these four indexes were determined.

**Conclusions:**

Four predictors for the comprehensive clinical risk and severity of coronary lesions of ACS were identified, which provided important information for the early diagnosis and appropriate treatment of ACS.

## Introduction

Coronary artery disease (CAD) is one of the most common cardiovascular diseases. As an acute CAD, acute coronary syndrome (ACS) is often caused by acute occlusion of coronary arteries by thrombosis from sudden rupture of atherosclerotic plaques. ACS is the most common cause of death in patients with CAD (1). It is estimated that about 661,000 people in the United States suffer from ACS in 2016 (2), and deaths from ACS occur at younger ages in low and middle income countries than in high income countries (3), which highlights the importance of risk assessment for ACS. Global Registry of Acute Coronary Events (GRACE) score is a risk assessment system for ACS based on risk factors summarized from clinical cases. It was recommended as one of the main methods for early risk assessment of ACS patients by the management guide for non-ST elevation myocardial infarction (NSTEMI) issued by the European Society of cardiology (ESC) in 2020 and the diagnosis and treatment guide for acute STEMI issued in 2017 (4, 5). However, the disadvantage of GRACE score is that it does not include the patient's coronary imaging information. The Synergy between Percutaneous Coronary Intervention with Taxus and Cardiac Surgery (SYNTAX) score is a scoring system for risk stratification based on the anatomical characteristics of coronary artery lesions, but with no clinical characteristics, which is commonly used to determine the complexity of CAD and identify high risk patients (6).

A few studies have assessed some indexes, such as homocysteine (7), complete blood count (8), serum albumin (9), and neutrophil to lymphocyte ratio (10) associated with severity or complexity of CAD as determined by the GRACE and SYNTAX scores in patients with ACS. However, most of these studies only evaluated the relationships between single indicators with GRACE or SYNTAX scores. The combination of these two scoring systems can consider both the clinical characteristics and coronary artery imaging characteristics of patients, which may more accurately describe the clinical status of patients to provide a more accurate prognosis. Identification of important indexes associated with clinical characteristics and coronary artery imaging characteristics may therefore improve the prognoses of patients with ACS by promoting early intervention, early vascular reconstruction, and appropriate treatment of percutaneous coronary intervention and coronary artery bypass graft surgery.

In this study, we investigated the relationships between clinical indexes and GRACE and SYNTAX scores, and identified the most important indexes predicting the severities of coronary lesions and comprehensive clinical risk in patients with ACS, and determined the important cut-off values of these indexes. Our findings provided important insight to be used for the early diagnoses and appropriate treatments of ACS patients.

## Materials and methods

### Study design and clinical data collection

Data of 225 ACS patients who underwent coronary angiography in the First Affiliated Hospital of Harbin Medical University during the period from September 2015 to November 2016 were collected. The inclusion criteria were as follows: (1) diagnosed with coronary angiography according to the management guide for non-ST elevation myocardial infarction (NSTEMI) issued by the ESC and the diagnosis and treatment guide for acute ST elevation myocardial infarction (STEMI) (4, 5); (2) no history of acute or chronic inflammatory disease; (3) no history of heart valve disease, congenital heart disease; and (4) no history of liver or kidney disease, autoimmune diseases, or tumors. The clinical data of the patients were collected, including age, sex, smoking, systolic blood pressure (SBP), diastolic blood pressure (DBP), heart rate, fasting blood glucose (FBG), total cholesterol (TC), triglyceride (TG), high density lipoprotein cholesterol (HDL-C), low density lipoprotein cholesterol (LDL-C), albumin, prealbumin, red blood cell distribution width (RDW), alanine aminotransferase (ALT), aspartate aminotransferase (AST), *γ*- Glutamyl aminotransferase (GGT), globulin, direct and indirect bilirubin, creatinine, N-terminal pro-brain natriuretic peptide (NT-proBNP), left ventricular ejection fraction (LVEF), and GRACE and SYNTAX scores were calculated. The study was approved by the Ethics Committee of the First Affiliated Hospital of Harbin Medical University (no. 201806) and was performed according to the principles of the Helsinki Declaration. Written informed consent was signed by all participants.

### Determination of biochemical indexes

Venous blood was collected after 12 h fasting for the assessment of RDW and serum concentrations of FBG, TC, TG, HDL-C, LDL-C, albumin, prealbumin, ALT, AST, GGT, bilirubin, creatinine and NT-proBNP. FBG was measured using the glucose oxidase method. TC, TG, HDL-C, LDL-C, albumin, prealbumin, ALT, AST, GGT, globulin, and bilirubin were measured using enzymatic methods. RDW was determined using Sysmex XN-1000 (Sysmex, Kobe, Japan), and other indicators were determined using an automatic biochemical analyzer (AU5400; Beckman Coulter, Pasadena, CA, USA).

### Blood pressure, heart rate, LVEF of heart and coronary angiography

Blood pressure, heart rate was determined using a multi-function monitor [DINAMAP PRO 1000, GE medical Systems (China) Co. Ltd., Wuxi, China]. LVEF of the heart was determined using Color Doppler echocardiography with a heart color ultrasonic detector (iE33; Philips, Cambridge, MA, USA). Coronary angiography was performed using an Innova IGS 520 (General Electric Health Care, Chalfont St. Giles, UK) and the angiographic images were analyzed by two experienced interventional cardiologist. The angiography was performed through the right radial artery using a 5F TIG tube. The left coronary angiography was in four positions: head position, foot position, spider position, and right shoulder position. The right coronary angiography was in two positions: the left anterior oblique position and head position. The parameters including degree of coronary stenosis, lesion length, and blood flow level were recorded.

### GRACE and SYNTAX scores

Eight items were included in the GRACE score: age, heart rate, systolic blood pressure, serum creatinine value, Killip classification of cardiac function, known cardiac events, myocardial enzyme markers, and ST segment changes of the electrocardiogram. The theoretical scores range from 2 to 383, and all indexes were collected immediately after the patient was admitted to the hospital. The syntax scoring system adopted the 16 segment method of a coronary tree to evaluate lesions of diameters ≥1.5 mm and stenosis of the coronary ≥50%, combining the dominant type of coronary artery, lesion location, stenosis degree, and characteristics of lesions. Its integral algorithm included 12 items, the first three of which were the dominant type of coronary artery, the number of lesions, and the number of segments of diseased vessels. The other nine items were the characteristics of lesions (complete occlusion, trifurcation, bifurcation, aorta, open lesions, serious tortuosity, length of lesions >20 mm, severe calcification, thrombus, and diffusion/small vessel lesions). The total score of each lesion after scoring was the syntax score. For multiple lesions in a segment, if the distance was less than three times the reference diameter, it was scored as one lesion, if the distance was more than three times the reference diameter, it was scored as two lesions. The syntax score represented the complexity of the coronary artery disease, and was used to guide the selection of reasonable means of revascularization (11).

### Statistical analysis

Statistical analyses were performed using SPSS statistical software for Windows, version 19.0 (SPSS, Chicago, IL, USA). Data are presented as the mean ± standard deviation (SD) for normally distributed data or as a median (interquartile range) for nonparamerically distributed data. The study subjects were divided into two groups according to the values of GRACE and SYNTAX scores. Differences in characteristics of ACS patients between the two groups were compared using the independent samples *t-*test for continuous variables, and with the χ2 test or Fisher's exact tests for categorical variables. Multiple logistic regression analysis (stepwise) was used to identify independent predictors of high GRACE and SYNTAX scores. The predictive ability of predictors, and model and area under the curve (AUC), were determined using receiver operating characteristics (ROC) analysis. The maximum value of the Youden index was used to determine the sensitivity and specificity. *P* < 0.05 was considered significant, and the values were two-tailed.

## Results

### Characteristics of ACS patients

A total of 225 ACS patients undergoing coronary angiography were enrolled in the study. The mean age was 64.66 ± 11.05 years (range: 34–88 years), 141 were male and 84 were female, and 57.3% of them were smokers. The mean values of SBP, DBP, heart rate, FBG, TC, TG, HDL-C, LDL-C, albumin, prealbumin, RDW, globulin, direct bilirubin, indirect bilirubin, LVEF, GRACE score, and SYNTAX score, as well as the median of ALT, AST, GGT, creatinine, and NT-proBNP are shown in Table 1.

**Table 1:** Characteristics of patients.

	Patients (*n* = 225)
Age (years)	64.66 ± 11.05
Male sex (*n*, %)	141 (62.7)
Smoking (*n*, %)	129 (57.3)
SBP (mmHg)	151.31 ± 22.89
DBP (mmHg)	84.20 ± 14.05
Heart rate (bpm)	77.12 ± 12.67
FBG (mmol/L)	6.42 ± 2.86
TC (mmol/L)	4.47 ± 1.22
TG (mmol/L)	1.86 ± 1.3
HDL-C (mmol/L)	1.13 ± 0.33
LDL-C (mmol/L)	2.99 ± 0.86
Albumin (g/L)	40.13 ± 4.72
Prealbumin (mg/dl)	19.13 ± 4.91
RDW (%)	11.75 ± 1.87
ALT (U/L)^a^	21.90 (14.10, 32.55)
AST (U/L)^a^	22.20 (17.55, 30.15)
GGT (U/L)^a^	28.00 (20.45, 47.75)
Globulin (g/L)	27.63 ± 4.35
Direct bilirubin (μmol/L)	3.19 ± 2.03
Indirect bilirubin (μmol/L)	12.23 ± 4.63
Creatinine (mmol/L)^a^	69.50 (82.10, 60.80)
NT-proBNP (ng/L)^a^	402.40 (928.00, 227.55)
LVEF (%)	58.96 ± 8.93
GRACE score	104.84 ± 33.07
SYNTAX score	18.74 ± 11.28

^a^ Data are expressed as median (interquartile range).

SBP, systolic blood pressure; DBP, diastolic blood pressure; bpm, beats per minute; FBG, fasting blood glucose; TC, total cholesterol; TG, triglyceride; HDL-C, high density lipoprotein cholesterol; LDL-C, low density lipoprotein cholesterol; RDW, red blood cell distribution width; ALT, alanine aminotransferase; AST, aspartate aminotransferase; GGT, *γ*- Glutamyl aminotransferase; NT-proBNP, N-terminal pro-brain natriuretic peptide; LVEF, Left ventricular ejection fraction. GRACE, Global Registry of Acute Coronary Events; SYNTAX, Synergy between Percutaneous Coronary Intervention with Taxus and Cardiac Surgery.

### Differences of indexes in ACS patients grouped by GRACE or SYNTAX scores

GRACE and SYNTAX were calculated from the clinical variables and imaging characteristics of coronary arteries. The relationships between indexes and comprehensive clinical risks and severities of coronary lesions were therefore analyzed using the GRACE or SYNTAX scores, respectively. ACS patients with a GRACE score ≥ 140 were assigned to the high GRACE score group (*n* = 42), while those with a GRACE score < 140 were assigned to the low GRACE score group (*n* = 183). When compared with patients in the low GRACE score group, patients in the high GRACE score group showed significantly higher levels of age, FBG, RDW, AST, direct bilirubin, creatinine, NT-proBNP, and lower levels of blood lipid, albumin, prealbumin, and LVEF (Table 2). ACS patients with a SYNTAX score ≥ 32 were assigned to the high SYNTAX score group (*n* = 52), while those with a SYNTAX score < 32 were assigned to the low SYNTAX score group (*n* = 173). Patients in the high SYNTAX score group showed higher levels of heart rate, FBG, LDL-C, RDW, NT-proBNP, and lower levels prealbumin and LVEF, when compared with patients in the low SYNTAX score group (Table 3).

**Table 2:** Comparison of indexes in different GRACE score groups.

	GRACE score < 140	GRACE score ≥ 140	*P*
	(*n* = 183)	(*n* = 42)	
Age (years)	61.96 ± 10.06	76.43 ± 6.55	<0.001
Male sex (*n*, %)	117 (83.0)	24 (17.0)	0.412
Smoking (*n*, %)	102 (79.1)	27 (20.9)	0.380
SBP (mmHg)	151.56 ± 21.59	150.19 ± 28.15	0.727
DBP (mmHg)	84.11 ± 12.74	84.55 ± 18.93	0.858
Heart rate (bpm)	76.91 ± 12.53	78.07 ± 13.38	0.592
FBG (mmol/L)	6.17 ± 2.05	7.53 ± 4.95	0.005
TC (mmol/L)	4.63 ± 1.22	3.78 ± 0.96	< 0.001
TG (mmol/L)	1.96 ± 1.4	1.41 ± 0.55	0.013
HDL-C (mmol/L)	1.16 ± 0.34	0.99 ± 0.24	0.003
LDL-C (mmol/L)	3.05 ± 0.88	2.72 ± 0.70	0.027
Albumin (g/L)	40.91 ± 4.33	36.73 ± 4.89	< 0.001
Prealbumin (mg/dl)	20.2 ± 4.49	14.46 ± 3.87	< 0.001
RDW (%)	11.62 ± 1.96	12.31 ± 1.31	0.032
ALT (U/L)^a^	21.60 (14.50, 31.30)	24.40 (11.95, 45.48)	0.423
AST (U/L)^a^	22.20 (17.60, 29.20)	22.55 (17.33, 57.73)	0.049
GGT (U/L)^a^	30.90 (21.20, 49.00)	21.40 (17.78, 35.25)	0.189
Globulin (g/L)	27.38 ± 4.33	28.72 ± 4.33	0.071
Direct bilirubin (μmol/L)	3.02 ± 1.80	3.92 ± 2.73	0.046
Indirect bilirubin (μmol/L)	12.22 ± 4.65	12.28 ± 4.6	0.937
Creatinine (mmol/L)	67.50 (60.20, 80.30)	74.00 (66.50, 90.78)	0.003
NT-proBNP (ng/L)	356.70 (188.00, 642.00)	1185.50 (524.30, 3293.75)	<0.001
LVEF (%)	59.97 ± 8.43	54.6 ± 9.84	<0.001

^a^ Data are expressed as median (interquartile range).

GRACE, Global Registry of Acute Coronary Events; SBP, systolic blood pressure; DBP, diastolic blood pressure; bpm, beats per minute; FBG, fasting blood glucose; TC, total cholesterol; TG, triglyceride; HDL-C, high density lipoprotein cholesterol; LDL-C, low density lipoprotein cholesterol; RDW, red blood cell distribution width; ALT, alanine aminotransferase; AST, aspartate aminotransferase; GGT, *γ*- Glutamyl aminotransferase; NT-proBNP, N-terminal pro-brain natriuretic peptide; LVEF, Left ventricular ejection fraction.

**Table 3:** Comparison of indexes in different SYNTAX score groups.

	SYNTAX score < 32	SYNTAX score ≥ 32	*P*
	(*n* = 173)	(*n* = 52)	
Age (years)	64.63 ± 11.68	64.77 ± 8.7	0.926
Male sex (*n*, %)	114 (80.9)	27 (19.1)	0.068
Smoking (*n*, %)	97 (75.2)	32 (24.8)	0.484
SBP (mmHg)	150.84 ± 23.02	152.87 ± 22.63	0.577
DBP (mmHg)	84.82 ± 15.02	82.13 ± 10.02	0.229
Heart rate (bpm)	76.13 ± 11.81	80.44 ± 14.85	0.031
FBG (mmol/L)	6.17 ± 2.30	7.27 ± 4.13	0.015
TC (mmol/L)	4.4 ± 1.13	4.72 ± 1.45	0.098
TG (mmol/L)	1.9 ± 1.43	1.73 ± 0.72	0.422
HDL-C (mmol/L)	1.13 ± 0.34	1.10 ± 0.32	0.458
LDL-C (mmol/L)	2.92 ± 0.8	3.2 ± 1.03	0.040
Albumin (g/L)	40.09 ± 4.69	40.25 ± 4.87	0.827
Prealbumin (mg/dl)	20.01 ± 4.66	16.19 ± 4.61	<0.001
RDW (%)	11.5 ± 1.92	12.56 ± 1.45	<0.001
ALT (U/L)^a^	22.30 (14.50, 32.55)	21.35 (12.65, 33.28)	0.792
AST (U/L)^a^	22.10 (17.30, 29.70)	25.35 (17.90, 33.33)	0.761
GGT (U/L)^a^	27.80 (20.05, 48.65)	28.00 (20.43, 42.15)	0.774
Globulin (g/L)	27.37 ± 4.23	28.49 ± 4.69	0.105
Direct bilirubin (μmol/L)	3.14 ± 1.80	3.35 ± 2.68	0.524
Indirect bilirubin (μmol/L)	12.37 ± 4.48	11.75 ± 5.11	0.403
Creatinine (mmol/L)	71.60 (61.65, 81.90)	65.40 (59.85, 82.88)	0.645
NT-proBNP (ng/L)	364.20 (177.20, 693.65)	637.20 (376.30, 1449.25)	<0.001
LVEF (%)	59.95 ± 8.57	55.69 ± 9.42	0.002

^a^ Data are expressed as median (interquartile range).

SYNTAX, Synergy between Percutaneous Coronary Intervention with Taxus and Cardiac Surgery; SBP, systolic blood pressure; DBP, diastolic blood pressure; bpm, beats per minute; FBG, fasting blood glucose; TC, total cholesterol; TG, triglyceride; HDL-C, high density lipoprotein cholesterol; LDL-C, low density lipoprotein cholesterol; RDW, red blood cell distribution width; ALT, alanine aminotransferase; AST, aspartate aminotransferase; GGT, *γ*- Glutamyl aminotransferase; NT-proBNP, N-terminal pro-brain natriuretic peptide; LVEF, Left ventricular ejection fraction.

### Identification of important indexes predicting severities of coronary lesions and comprehensive clinical risks of ACS patients

Multiple logistic regression analysis with stepwise selection was used for screening the important predictors of comprehensive clinical risk and severity of coronary lesions using the GRACE or SYNTAX scores. Table 4 shows that AST was positively associated with the GRACE score, and TC and RDW were positively associated with the SYNTAX score. AST was important predictors of comprehensive clinical risks, and TC and RDW were important predictors of the severities of coronary lesions, which were risk factors for ACS patients. Prealbumin was negatively associated with the GRACE score and also with the SYNTAX score, and was an important predictor of both comprehensive clinical risks and the severities of coronary lesions, which were protective factors for ACS patients.

**Table 4:** Multivariate logistic analyses for important predictors of comprehensive clinical risk and severity of coronary lesions through GRACE and SYNTAX scores in ACS patients.

	B	SE	Wald	OR	95% CI	*P*
**GRACE**						
Prealbumin	−0.298	0.051	33.875	0.743	0.672–0.821	<0.001
AST	0.011	0.005	5.985	1.011	1.002–1.021	0.014
**SYNTAX**						
Prealbumin	−0.179	0.042	17.847	0.836	0.769–0.909	<0.001
TC	0.416	0.142	8.582	1.517	1.148–2.004	0.003
RDW	0.422	0.135	10.747	1.556	1.195–2.028	0.001

GRACE, Global Registry of Acute Coronary Events; SYNTAX, Synergy between Percutaneous Coronary Intervention with Taxus and Cardiac Surgery; AST, aspartate aminotransferase; TC, total cholesterol; RDW, red blood cell distribution width.

### Predictive ability of predictors and models predicting the severities of coronary lesions and comprehensive clinical risks of ACS patients

**Figure 1:**
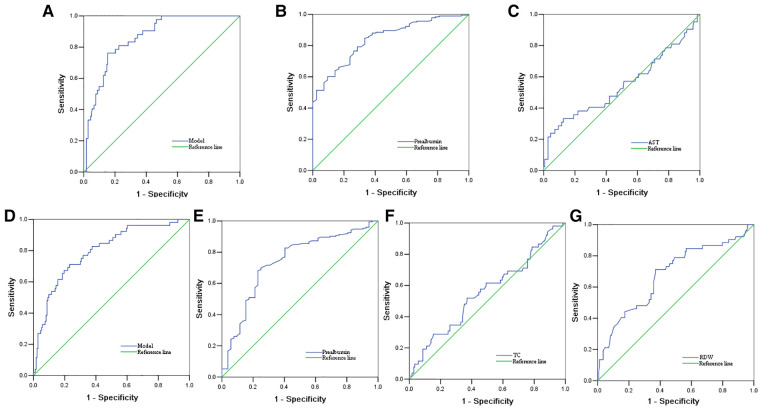
Receiver operating characteristic curves. **A–C**, The model (**A**) and included variables of prealbumin (**B**) and aspartate aminotransferase (AST) (**C**) in the prediction of comprehensive clinical risks. **D–G**, The model (**D**) and included variables of prealbumin (**E**), total cholesterol (TC) (**F**), and red blood cell distribution width (RDW) (**G**) in the prediction of coronary lesion severities.

ROC analyses were performed on important predictors and models for predicting comprehensive clinical risks and severities of coronary lesions using GRACE or SYNTAX scores of ACS patients. Table 5 and the Figure 1 show the results of ROC analyses. The AUC, sensitivity, and specificity based on estimates of the logistic model showed that the two models for high GRACE or SYNTAX scores fit very well, and had the highest predictive efficacy using the combination index of prealbumin and AST in the high GRACE score risk factor regression mode, and prealbumin, TC, and RDW in the high SYNTAX score risk factor regression mode (AUC = 0.862, sensitivity = 81%, specificity = 78% for GRACE; AUC = 0.797, sensitivity = 71%, specificity = 77% for SYNTAX scores). The AUCs for prealbumin and AST were 0.840 (95% CI: 0.738–0.898) and 0.547 (0.437–0.657), respectively, and the AUCs for prealbumin, TC, and RDW were 0.739 (0.660–0.818), 0.557 (0.465–0.649), and 0.678(0.591–0.765), respectively. The optimal cut-off values of prealbumin and AST for comprehensive clinical risk were 17.25 mg/dl and 35.90 U/L, respectively, and for the severities of coronary lesions, the optimal cut-off values of prealbumin, TC, and RDW were 18.15 mg/dl, 4.64 mmol/L, and 12.09%, respectively.

**Table 5:** Receiver operator characteristic analyses of important indexes and model in ACS patients.

	AUC (95% CI)	Best cutoff	Sensitivity (%)	Specificity (%)	Maximum of Youden index^a^
**GRACE score**					
Model	0.862 (0.809–0.915)	0.22^b^	81	78	0.59
Prealbumin (mg/dl)	0.840 (0.783–0.898)	17.25	79	71	0.51
AST (U/L)	0.547 (0.437–0.657)	35.90	33	86	0.20
**SYNTAX score**					
Model	0.797 (0.728–0.866)	0.27^b^	71	77	0.48
Prealbumin (mg/dl)	0.739 (0.660–0.818)	18.15	70	75	0.45
TC (mmol/L)	0.557 (0.465–0.649)	4.64	52	63	0.15
RDW (%)	0.678 (0.591–0.765)	12.09	69	63	0.32

^a^ Sensitivity + specificity – 1.

^b^ P of Logistic.

AUC, area under the curve; GRACE, Global Registry of Acute Coronary Events; SYNTAX, Synergy between Percutaneous Coronary Intervention with Taxus and Cardiac Surgery; AST, aspartate aminotransferase; TC, total cholesterol; RDW, red blood cell distribution width.

## Discussion

The current study showed that high AST could predict a comprehensive clinical risk, and high total cholesterol and RDW could predict the severity of coronary lesions. Low prealbumin could predict both severity of coronary lesions and comprehensive clinical risks of ACS. A model with a combination of prealbumin and AST had the highest predictive efficacy for comprehensive clinical risks, and a combination of prealbumin, TC, and RDW had the highest predictive efficacy for the severities of coronary lesions.

ACS, including STEMI, NSTEMI and unstable angina, can occur in patients with ischemic heart disease, which are major causes of death worldwide, especially in the elderly (4, 12). AST was positively related to GRACE as a risk factor of ACS patients in the present study. AST, a pyridoxal phosphate dependent transaminase, is a well-known marker for liver injury. It can be expressed in both liver and myocardial tissues (13, 14). In healthy myocardial tissue, AST was present in cells, but it normally only exists in low levels in the serum. However, when myocardial hypoxia occurs, the permeability of cell membranes changes, and AST in cytoplasm releases into the blood, resulting in an increase of AST. AST can be further increased due to myocardial cell necrosis, mitochondrial damage, and disintegration (15). Shen et al. (15) reported that coronary heart disease (CHD) patients had higher levels of serum AST, when compared with subjects with no CHD, and high serum AST was a biochemical marker to predict the severity of CHD, and an independent risk factor for CHD. Serum AST level elevated in patients with ACS (16), and was significantly associated with long-term mortality after acute myocardial infarction (17). In addition, increased AST was closely correlated with nonalcoholic steatohepatitis (18), obesity (19), diabetes (20), and skeletal muscle disease (21). Because so many factors can cause an increase of AST levels, an increased level will usually require further diagnostic studies. It is therefore significant that a combination of AST and other important indexes obtained in the present study can be used for evaluating the risk of ACS, prognosis, and treatment guidance.

In the present study, TC was positively associated with coronary artery disease severity. This was consistent with earlier studies that reported an important role of TC in assessing the severity of myocardial infarction (22), and its significantly direct association with the higher probability (23) and mortality in CHD (24, 25) and high CVD risk (26). A study by Bjorck et al. (25) reported that, in Swedish patients between 1986 and 2002, more than half of the decrease in coronary heart disease mortality was mainly attributed to the reduction of cardiovascular risk factors, mainly a significant decrease in TC. Such decreases in cholesterol levels could be achieved by statin medication (27) or lifestyle management, such as diet control, increased physical activity, and weight control (25, 28–30).

In the present study, RDW was also found to be positively associated with the severity of CAD as a risk factor in ACS patients. RDW is a parameter reflecting the heterogeneity of red blood cell volume, which is expressed by the coefficient of variation of red blood cell volume. It is more objective and accurate than the observations of uneven shape and size of red blood cells using a blood smear. The basic pathogenesis of ACS is the formation, rupture, and thrombosis of coronary atherosclerotic plaques. Atherosclerosis is also an inflammatory process that responds to various risk factors (31), whose development is closely related to chronic inflammation of the arterial wall (32). The inflammatory state, oxidative stress, and neuroendocrine activation state during the occurrence of ACS can cause tumor necrosis factor-α (TNF-α) and interleukin-6 (IL-6) to inhibit the precursor of erythrocytes, prevent the formation of erythrocytes, promote apoptosis of erythrocytes, and release immature red blood cells into the blood, which result in an increase of size heterogeneity of peripheral blood red blood cells, leading to an increase of RDW (33). Tunçez et al. (34) reported that RDW values higher than 13.9 could predict the risk for the development of ACS with stent thrombosis in STEMI patients undergoing emergency PCI, with a sensitivity and specificity of 57% and 52%, respectively. It suggested that RDW was associated with the severity and prognosis of ACS. Our study provided further evidence for this finding. In the present study, prealbumin was found to be strongly associated with ACS. Prealbumin is a negative reactive protein of the acute-phase, is mainly synthesized and secreted by the liver, and plays an important role in physiological processes such as the stress response, necrotic material clearance, and tissue repair (35). Because its biological half-life is significantly shorter than that of albumin, it is more sensitive than albumin in evaluating the magnitude of inflammatory reactions, degree of liver function damage, and malnutrition (36, 37). Moreover, the occurrence and progression of coronary atherosclerotic lesions involve an inflammatory reaction, endothelial dysfunction, oxidative stress, platelet activation, and other mechanisms (38–40). Because prealbumin is closely related to the above pathological processes, the relationship between prealbumin and atherosclerotic disease was determined. In the present study, we found that prealbumin was negatively associated with the GRACE and SYNTAX scores. The GRACE risk score is a scoring model based on a variety of clinical variables, while the SYNTAX scoring system is based on the imaging characteristics of coronary arteries. Low prealbumin may therefore predict a higher clinical comprehensive risk and severity of coronary artery disease. In addition, the combined application of these four indexes had better predictive ability. Limitations to this study included that although many indexes were included in the study, some clinical biochemical indexes were not included in this study because of incomplete data or experimental conditions. These indexes might be meaningful for our research. In addition, a large sample size is important for risk factor analysis of disease, and this study was a single-center retrospective study. Further studies with larger samples from multiple centers are needed to verify our research results.

In conclusion, in the present study, four important predictors for the severity of coronary lesions and comprehensive clinical risks of AC were identified, and their important cut-off values were determined. These findings might provide valuable information for the early diagnosis and appropriate treatments for ACS patients.

## Data Availability

The raw data supporting the conclusions of this article will be made available by the authors, without undue reservation.
